# Effect of cyclic bis(3′–5′)diguanylic acid and its analogs on bacterial biofilm formation

**DOI:** 10.1111/j.1574-6968.2009.01825.x

**Published:** 2009-12

**Authors:** Yuka Ishihara, Mamoru Hyodo, Yoshihiro Hayakawa, Taichi Kamegaya, Keiko Yamada, Akira Okamoto, Tadao Hasegawa, Michio Ohta

**Affiliations:** 1 Department of Bacteriology, Graduate School of MedicineNagoya, Japan; 2 Graduate School of Information Science/Human Informatics and CREST of JST, Nagoya UniversityNagoya, Japan; 3 Department of Infection and Prevention Medicine, Nagoya City University Graduate School of Medical ScienceNagoya, Japan

**Keywords:** biofilm, cyclic-di-GMP, *Staphylococcus aureus*, *Pseudomonas aeruginosa*, regulation of biofilm formation, GdpS

## Abstract

Cyclic bis(3′–5′)diguanylic acid (cyclic-di-GMP) functions as a second messenger in diverse species of bacteria to trigger wide-ranging physiological changes. We measured cyclic-di-GMP and its structural analogs such as cyclic bis(3′–5′)guanylic/adenylic acid (cyclic-GpAp), cyclic bis(3′–5′)guanylic/inosinic acid (cyclic-GpIp) and monophosphorothioic acid of cyclic-di-GMP (cyclic-GpGps) for effects on the biofilm formation of *Staphylococcus aureus* and *Pseudomonas aeruginosa*. We constructed a knockout mutant of SA0701, which is a GGDEF motif protein relevant to diguanylate cyclase from *S. aureus* 2507. We confirmed that the biofilm formation of this mutant (MS2507ΔSA0701) was reduced. Cyclic-di-GMP corresponding to physiological intracellular levels given in the culture recovered the biofilm formation of MS2507ΔSA0701, whereas its analogs did not, indicating that unlike a previous suggestion, cyclic-di-GMP was involved in the positive regulation of the biofilm formation of *S. aureus* and its action was structurally specific. At a high concentration (200 μM), cyclic-di-GMP and its analogs showed suppression effects on the biofilm formation of *S. aureus* and *P. aeruginosa*, and according to the quantification study using costat analysis, the suppression potential was in the order of cyclic-di-GMP, cyclic-GpGps, cyclic-GpAp and cyclic-GpIp, suggesting that the suppression effect was not strictly specific and the change of base structure quantitatively affected the suppression activity.

## Introduction

Cyclic bis(3′–5′)diguanylic acid (cyclic-di-GMP or c-di-GMP) was discovered as an allosteric regulator of cellulose biosynthesis in *Gluconacetobacter xylinus* (*Acetobacter xylinum*) (Ross *et al.*, 1987). Recent research has accumulated evidence indicating that cyclic-di-GMP is a novel second messenger molecule of bacteria and regulates cell surface events such as biofilm formation and motility. Proteins carrying the domains GGDEF and EAL or HD-GYP are relevant to the activities of diguanylate cyclase and phosphodiesterase, respectively, and are involved in the metabolism of cyclic-di-GMP (Tal *et al.*, 1998; Ryan *et al.*, 2006;). Proteins carrying the GGDEF domain are almost ubiquitous in bacterial genomes except for only limited kinds of bacteria (Römling *et al.*, 2005; Schmidt *et al.*, 2005;), suggesting that cyclic-di-GMP is a universal messenger molecule in bacteria. Intracellular cyclic-di-GMP promotes biofilm formation and suppresses motility at elevated levels of concentration (Jenal & Malone, 2006; Kim & McCarter, 2007;), whereas extracellular cyclic-di-GMP of high concentrations suppressed the biofilm formation (Karaolis *et al.*, 2005).

Bacterial biofilm on the surface of medical devices is a major cause of hospital-associated infections (Lindsay & von Holy, 2006; Wenzel, 2007;). The control of the biofilm formation, therefore, is a prerequisite for the prevention of biofilm-associated infections. Compounds related in structure to cyclic-di-GMP are expected to have inhibitory activities on bacterial biofilm formation (Kline *et al.*, 2008). Moreover, the activities of these analogs will give us the insight into the structural specificity of the target molecules of cyclic-di-GMP. Cyclic-di-GMP can be bound by some diguanylate cyclase enzymes to allosterically repress its own synthesis (Chan *et al.*, 2004; Wassmann *et al.*, 2007;). The other known protein targets are *G. xylinus* cellulose synthase and other PilZ domain proteins (Ross *et al.*, 1985, 1987; Ryjenkov *et al.*, 2006), and *Pseudomonas aeruginosa* PelD protein (Lee *et al.*, 2007). It was found in a recent study that cyclic-di-GMP in many bacterial species is sensed by a riboswitch class in mRNA that controls the expression of genes involved in numerous fundamental cellular processes (Sudarsan *et al.*, 2008). Although the control mechanisms by cyclic-di-GMP for the development of *Staphylococcus aureus* and *P. aeruginosa* biofilms are not well understood and even irrelevant in the biofilm development of *S. aureus* as suggested in a recent report (Holland *et al.*, 2008), the question whether riboswitches that sense cyclic-di-GMP are involved in the regulatory pathway of cyclic-di-GMP remains to be solved.

Hayakawa *et al.* (2003) developed a new method for the chemical synthesis of cyclic-di-GMP in a high yield. In the course of systematic investigation on the bioactivity of cyclic-di-GMP-related compounds, they developed a synthetic method for a cyclic-di-GMP derivative cyclic bis(3′–5′)-2′-deoxyguanylic/guanylic acid (cyclic-dGpGp) and found that cyclic-dGpGp expressed a moderate suppression effect on the bacterial biofilm formation as well as weak repression on the bacterial motility, as compared with cyclic-di-GMP (Mano *et al.*, 2007). They also synthesized other analogs including analogs with modified nucleoside bases or internucleotide bonds (Hyodo *et al.*, 2006). The present study was undertaken using cyclic-di-GMP and its analogs to determine the structural specificity of recognition of the targets that sense cyclic-di-GMP in the positive regulation or negative regulation of the bacterial biofilm formation.

## Materials and methods

### Cyclic-di-GMP and its analogs

**Fig. 1:**
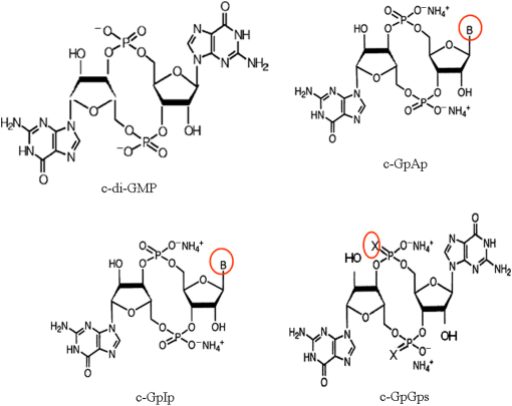
Structure of c-di-GMP and its analogs. B in c-GpAp represents adenine, B in c-GpIp represents inosine, and X in c-GpGps represents S.

Cyclic-di-GMP and its analogs were chemically synthesized as described previously (Hayakawa *et al.*, 2003; Hyodo *et al.*, 2006;). Cyclic-di-GMP analogs used in this study were cyclic bis(3′–5′)guanylic/adenylic acid (cyclic-GpAp or c-GpAp), cyclic bis(3′–5′)guanylic/inosinic acid (cyclic-GpIp or c-GpIp) and monophosphorothioic acid of cyclic-di-GMP (cyclic-GpGps or c-GpGps) (Hyodo *et al.*, 2006). The structures of these compounds are shown in Fig. 1. These compounds were dissolved in 0.85% NaCl for use.

### Bacterial strains and culture conditions

*Pseudomonas aeruginosa* strain PAO1 and *S. aureus* strain MS2507 were used in this study. *Staphylococcus aureus* MS2507 was isolated from the blood culture of a patient and was a highly biofilm-forming strain. These strains were cultured in tryptic soy broth (TSB), Luria–Bertani (LB; 1% Bacto tryptone, 0.5% Bacto yeast extract, pH 7.2% and 1.0% NaCl) or on LB agar plates overnight at 37 °C before use.

### Construction of an SA0701-deficient mutant

Based on the *S. aureus* N315 genome database, the sequence of SA0701 (Bfd1, GdpS), which carries the GGDEF motif, was identified (Tu Quoc *et al.*, 2007; Holland *et al.*, 2008;). A 0.65-kb N-terminal fragment of the gene for SA0701 was amplified with a forward primer TGCACTGCAGACCATTTATTCACCTTCATC and a reverse primer GGGGTACCATAACAATCAACGTAACACC, and a 0.72-kb C-terminal fragment was amplified with a forward primer ACGCGTCGACAAGGGCGAAACAAAGTAATG and a reverse primer ACGCGTCGACAAGGGCGAAACAAAGTAATG. The two PCR products were digested with the relevant restriction enzymes, and the N-terminal fragment was inserted into the PstI–KpnI site and the C-terminal fragment was inserted into the SalI–HindIII site of pYT3 (kindly provided by Hiramatsu, Juntendo University, Tokyo). The resulting plasmid pYT3ΔSA0701 was transformed into *S. aureus* MS2507 as described previously (Schen & Laddaga, 1992). The transformants were selected with the tetracycline resistance phenotype at 42 °C to isolate a ΔSA0701 mutant through a double-crossover recombination. The inactivation of the gene for SA0701 was verified using PCR, followed by DNA sequencing (data not shown).

### Quantification of biofilm formation

Quantification of biofilm formation by MS2507, MS2507ΔSA0701 and PAO1 was performed using a crystal violet staining method (Karaolis *et al.*, 2005) with some modifications. Bacteria were precultured on LB agar plates at 37 °C overnight. Culture conditions for biofilm formation were LB for PAO1 and TSB for MS2507 and MS2507ΔSA0701 at 30 °C 12-h static culture. The media for biofilm formation were supplemented with various concentrations of synthetic cyclic-di-GMP and its analogs. A single colony was inoculated into 4 mL of LB or TSB and incubated at 37 °C with constant shaking at 160 r.p.m. until late logarithmic stage. The culture was then diluted 1 : 10^4^ with fresh LB or TSB supplemented with various concentrations of cyclic-di-GMP and its analogs and a 200 μL of the diluted cultures was transferred into round-bottom wells of a polystyrene microtiter plate (Iwaki, Tokyo, Japan). The microtiter plate was incubated statically at 30 °C for 12 h. The supernatant was then discarded and the wells were washed three times with 200 μL of phosphate-buffered saline. After the wells were dried, 200 μL of 0.1% crystal violet was added and the wells were stained at room temperature for 15 min. The wells were then washed three times with distilled water to remove the extra dye and dried. The dye staining the biofilm in each well was extracted with 200 μL of dimethyl sulfoxide and the OD_570 nm_ was measured with a plate reader (Tecan Austria GmbH, Austria). These experiments were repeated at least three times, and the results were expressed as the mean values ± SD. *P*-values for significance were calculated by the Student *t*-test.

### Confocal laser scanning microscopic analysis of biofilm and comstat analysis of resultant images

PAO1 and MS2507 were prepared in the same way as those described in the quantification assay of the biofilm. Freshly prepared bacterial solutions were inoculated into 2 mL of LB for PAO1 and TSB for MS2507 supplemented with various concentrations of cyclic-di-GMP or its analogs in glass-based dishes (coverslip diameter 27 mm, Iwaki) and were incubated statically at 30 °C for 15 h for PAO1 and 9 h for MS2507. The culture supernatant was discarded and the dishes were carefully rinsed three times with 0.85% NaCl and viability staining of unfixed biofilms was performed using BacLight Live/Dead kit (L-7012, Molecular Probes) according to the manufacturer's recommendations to visualize biofilms. The biofilms attached on the polyethyleneimine-coated glass dishes (Iwaki) were observed at × 400 magnification using a confocal laser scanning microscope (LSM5 Pascal, Carl Zeiss Co. Ltd). Images were acquired at a 1-μm interval through the biofilms and five image stacks, each representing a different field of view, and were compiled from at least two independent experiments. The image stacks were analyzed using the comstat program (Heydorn *et al.*, 2000) and the following parameters were obtained: total biomass; mean thickness, the mean height of the biofilm; maximum thickness; roughness coefficient, a measure of how much the thickness of the biofilm varies; and surface-to-volume ratio, an estimate of the portion of the biofilm exposed to nutrients.

## Results

### The activation effect of cyclic-di-GMP and its analogs on *S. aureus* biofilm formation

A previous study showed that among a comprehensive collection of biofilm-defective mutants, a *bfd1* (*SA0701*)-knockout mutant was identified (Tu Quoc *et al.*, 2007). The gene *bfd1* encodes a hypothetical protein that contains a GGDEF motif. Holland *et al.* (2008), however, recently suggested that *Staphylococcus epidermidis* and *S. aureus* GdpS (same as SA0701) regulated staphylococcal biofilm formation independently of cyclic-di-GMP, although direct evidences such as the measurements of intracellular cyclic-di-GMP were not presented.

**Fig. 2:**
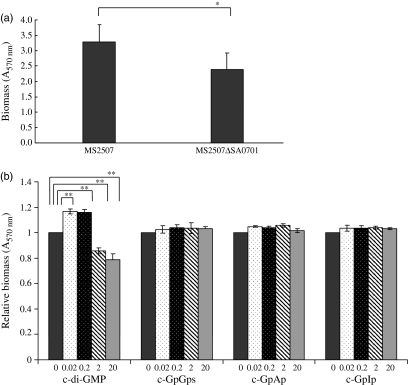
Biofilm formation of MS2507 and MS2507ΔSA0701, and effects of cyclic-di-GMP and its analogs. (a) Biofilm formation at 30°C 12 h of MS2507 and MS2507ΔSA0701 in TSB was measured using the crystal violet staining method. The biofilm formation of MS2507ΔSA0701 was moderately lower than that of the parent. (b) Biofilm formation of MS2507ΔSA0701 was measured by the presence of cyclic-di-GMP and its analogs. Only cyclic-di-GMP at low concentrations enhanced the biofilm formation of MS2507ΔSA0701. ^*^P<0.05; ^**^P<0.01.

We also found in the staphylococcal whole-genome database that SA0701 was the only suspected protein containing a GGDEF motif relevant to the biosynthesis of cyclic-di-GMP. We thus studied the effects of cyclic-di-GMP and its analogs at low doses on the biofilm formation of a SA0701 (GdpS)-deficient mutant to see whether these compounds could influence the staphylococcal biofilm formation. We constructed an SA0701-deficient mutant MS2507ΔSA0701 from *S. aureus* MS2507, a biofilm-positive wild strain, using a gene-targeting method. In a preliminary study, there was no difference in the growth rate in the culture broth used for the biofilm formation between MS2507 and MS2507ΔSA0701 (data not shown), suggesting that the inactivation of GdpS did not affect the growth of *S. aureus*. As Fig. 2 shows, we found that MS2507ΔSA0701 formed a reduced level of biofilm as compared with its parent and that 0.02–0.2 μM of cyclic-di-GMP enhanced the biofilm formation of MS2507ΔSA0701, although the level of biofilm was not fully recovered to that of the parent (data not shown). We therefore suggest that cyclic-di-GMP is involved in the regulation of the *S. aureus* biofilm formation, which is impaired by the loss of the function of GdpS. Interestingly, higher concentrations (2–20 μM) of cyclic-di-GMP suppressed the biofilm formation of MS2507ΔSA0701. We also found that none of the cyclic-di-GMP analogs showed positive regulatory effects on the biofilm formation of MS2507ΔSA0701.

### Suppression effect of cyclic-di-GMP analogs at high concentrations on biofilm formation

**Fig. 3:**
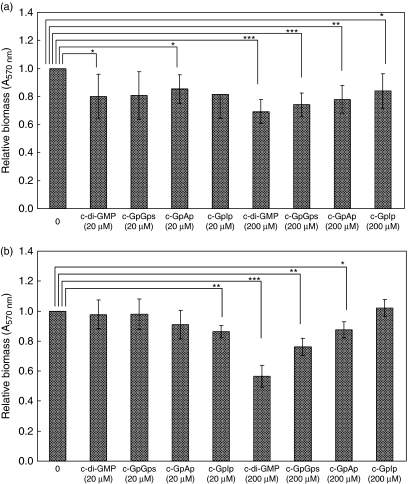
Quantitative analysis of the suppressive effect of cyclic-di-GMP and its analogs on *Staphylococcus aureus* biofilm formation (a) and on *Pseudomonas aeruginosa* biofilm formation (b) using the crystal violet staining method. The biofilm of *S. aureus* MS2507 and *P. aeruginosa* PAO1 was formed on wells of a polystyrene microplate. The media for biofilm formation were supplemented with indicated concentrations of synthetic cyclic-di-GMP and its analogs. After the incubation, the biofilm formed on the surface of wells was stained with crystal violet. The dye staining the biofilm was then extracted and the OD_570 nm_ was measured. Relative values of each culture to the control culture containing none of the synthetic cyclic-di-GMP and its analogs are presented in the figure. ^*^P<0.05; ^**^P<0.01; ^***^P<0.001.

Both gram-negative *P. aeruginosa* PAO1 and gram-positive *S. aureus* MS2507 form biofilms on the surfaces of polystyrene wells and polyethyleneimine-coated glass dishes. The effects of three analogs, cyclic-GpAp, cyclic-GpIp and cyclic-GpGps, on the formation of the biofilm on the polystyrene surface by these bacteria were measured. All of the three analogs and cyclic-di-GMP suppressed the biofilm formation of MS2507 at 200 μM (Fig. 3). These compounds were suppressive even at a low concentration (20 μM), although the effects were not high. Similar suppressive effects were observed on the biofilm formation of *P. aeruginosa* PAO1 when cyclic-GpGps, cyclic-GpAp and cyclic-di-GMP were added at a concentration of 200 μM (Fig. 3). On the other hand, these compounds did not show a significant suppressive effect at 20 μM. Cyclic-GpIp suppressed the biofilm formation of PAO1 at a low concentration but not at a high concentration. Their suppressive effects at 200 μM on the biofilm formation of both *S. aureus* and *P. aeruginosa* were high in the order of cyclic-di-GMP, cyclic-GpGps, cyclicGpAp and cyclic-GpIp.

Similar results were obtained when these compounds were measured for the effects on the biofilm formation of MS2507 and PAO1 with a confocal laser scanning microscopy followed by the quantification using the comstat program. Most of the bacterial cells within the biofilms of MS2507 and PAO1 in the presence of cyclic-di-GMP and its analogs were alive as measured by qualitative viability staining using the BacLight Live/Dead kit (data not shown). This indicates that the suppressive effects of these compounds on the biofilm formation were not caused by the killing of bacteria. This was also confirmed with the growth kinetics of MS2507 and PAO1 in a rich medium in the presence of these compounds at 200 μM (data not shown). The comstat analysis presented the scores of the total biomass, average thickness, maximum thickness, roughness and surface/volume ratio for the MS2507 biofilms in Table 1 and for the PAO1 biofilms in Table 2. Biofilms of MS2507 and of PAO1 formed in the presence of cyclic-di-GMP and cyclic-GpGps at 200 μM had significantly lower total biomass, average thickness and maximum thickness than those formed in the absence of the cyclic nucleotide compounds. The suppressive effect of cyclic-GpGps was almost similar to that of cyclic-di-GMP. On the other hand, cyclic-GpAp showed only a moderate suppressive effect and cyclic-GpIp did not show a clear suppressive effect on the biofilm formation of MS2507 and PAO1. The scores of roughness and surface/volume for the MS2507 biofilms and PAO1 biofilms treated with cyclic-di-GMP and cyclic-GpGps were much higher than those for the untreated biofilms, suggesting that the biofilms suppressed by these compounds were rather rough and sparse.

**Table 2:** Quantitative analysis of PAO1 biofilm formed in the presence of cyclic-di-GMP and its analogs

Materials	Biomass (μm^3^ μm^−2^)	Average thickness (μm)	Maximum thickness (μm)	Roughness coefficient	Surface/volume ratio
c-di-GMP	0.444 ± 0.193***	0.223 ± 0.158***	5.400 ± 0.548***	1.681 ± 0.191	3.303 ± 0.206
c-GpGps	0.453 ± 0.125***	0.273 ± 0.131***	4.600 ± 1.140***	1.677 ± 0.095	3.549 ± 0.240
c-GpAp	1.054 ± 0.595*	0.773 ± 0.493**	6.400 ± 1.140***	1.298 ± 0.354	2.858 ± 0.356
c-GpIp	2.618 ± 0.451*	2.343 ± 0.570	11.167 ± 2.483**	0.638 ± 0.091	2.105 ± 0.125
No treatment	2.098 ± 0.297	1.944 ± 0.438	16.200 ± 0.837	0.967 ± 0.095	2.300 ± 0.107

The analysis of image stacks was conducted using the comstat program. Results represent the mean ± SD and *P*-values refer to comparison with no treatment biofilm.

* *P*<0.05.

** *P*<0.01.

*** *P*<0.001.

**Table 1:** Quantitative analysis of MS2507 biofilm formed in the presence of cyclic-di-GMP and its analogs.

Materials	Biomass (μm^3^ μm^−2^)	Average thickness (μm)	Maximum thickness (μm)	Roughness coefficient	Surface/volume ratio
c-di-GMP	0.220 ± 0.056*	0.011 ± 0.003*	1.250 ± 0.500***	1.978 ± 0.012	3.639 ± 0.494
c-GpGps	0.229 ± 0.001*	0.015 ± 0.012*	1.250 ± 0.500***	1.970 ± 0.024	3.605 ± 0.469
c-GpAp	1.184 ± 0.159	0.704 ± 0.170	4.500 ± 0.577	1.129 ± 0.087	2.153 ± 0.289
c-GpIp	1.614 ± 0.289	1.008 ± 0.308	4.000 ± 0.000	0.750 ± 0.093	1.714 ± 0.116
No treatment	1.735 ± 0.548	1.143 ± 0.507	3.750 ± 0.957	0.913 ± 0.109	1.802 ± 0.239

The analysis of image stacks was conducted using the comstat program. Results represent the mean ± SD and *P*-values refer to comparison with no treatment biofilm.

* *P*<0.05.

*** *P*<0.001.

## Discussion

The present study exhibited that the target molecules of cyclic-di-GMP that were involved in the biofilm formation recognized the difference of the base structure of cyclic nucleotides as well as the monophosphorothioic acid structure. At low concentrations, cyclic-di-GMP enhanced *S. aureus* biofilm formation, which was reduced by the inactivation of the GdpS gene, whereas none of the analogs examined in this study showed such activity. The concentration of cyclic-di-GMP in the culture to show positive regulation for the biofilm formation ranged 0.02–0.2 μM, which was similar to the estimated intracellular concentration of cyclic-di-GMP in normal bacterial cells (Simm *et al.*, 2009). A previous report showed that the GGEEF (GGDEF) motif of GdpS essential for diguanylate cyclase activity was not functional and that GdpS controlled biofilm development via a cyclic-di-GMP-independent mechanism (Holland *et al.*, 2008). On the other hand, we found that cyclic-di-GMP functioned to recover the *S. aureus* biofilm formation, which was impaired by the inactivation of GdpS, suggesting that cyclic-di-GMP can be involved in the regulation of *S. aureus* biofilm formation. Although, in a recent study, the *gdpS* mutant displayed no difference in the biofilm formation capacity compared with the wild type (Shang *et al.*, 2009), the *gdpS* mutant constructed in this study and a transposon-inserted mutant of *gdpS* isolated from *S. aureus* S30 had an impaired biofilm formation capacity (Tu Quoc *et al.*, 2007). It may possibly be due to physiological differences among the strains, because strains MS2507 and S30 exhibited a strong biofilm-forming phenotype, whereas NCTC8325, which was used as the parent by Shang, did not. It should be noted that in our preliminary study the impaired biofilm formation capacity of MS2507ΔSA0701 was complemented by the introduction of an intact *gdpS* gene on a plasmid. Because the activation effect of extrinsic cyclic-di-GMP on the biofilm formation was observed in a GdpS-deficient mutant, it is unlikely that GdpS is the sole receptor of cyclic-di-GMP in the regulation of *S. aureus* biofilm formation. The presence of other targets similar to previously reported proteins such as PilZ domain proteins, PelD and FleQ, may also be possible although bioinformatics analysis indicated the absence of such proteins in *S. aureus*. Our results would provide a clue for identification of new cyclic-di-GMP signaling pathways in *S. aureus* biofilm formation.

On the other hand, high doses of cyclic-di-GMP and the analogs suppressed biofilm formation of *S. aureus* and *P. aeruginosa*, suggesting that the recognition specificity of the relevant targets for the suppression of the bacterial biofilm formation was relatively lower than those for the positive regulation. It should be noted again that the suppressive concentrations of cyclic-di-GMP and its analogs added in the culture were 100–1000 times higher than the estimated physiological intracellular levels of cyclic-di-GMP (Simm *et al.*, 2009). Previous studies demonstrated that intracellular cyclic-di-GMP produced by the overexpressed biosynthesis genes promoted biofilm formation in *P. aeruginosa* (Kulasakara *et al.*, 2006). In the report, intracellular cyclic-di-GMP produced by overexpression of the biosynthesis genes was supposed to be a high level comparable to the concentration of cyclic-di-GMP added in the culture, but the biofilm formation in overexpressed *P. aeruginosa* cells was not suppressed. The concentration of intracellular cyclic-di-GMP in overexpressed cells, however, was not presented in these reports. The concentration of intracellular cyclic-di-GMP should be affected by the concentration of GTP, the substrate of diguanylate cyclase and by phosphodiesterase hydrolysis. We therefore speculate that the concentration of intracellular cyclic-di-GMP in overexpressed *P. aeruginosa* cells may be much lower than the suppressive concentration of cyclic-di-GMP added in the culture.

Because the suppression potential of cyclic-GpGps was similar to that of cyclic-di-GMP whereas those of cyclic-GpAp and cyclic-GpIp were much lower, the substitution of the base structure rather than the replacement of oxygen with sulfur in phosphate, which changes the electronic state in the cyclic structure, would affect the suppression activity of cyclic-di-GMP.

Cyclic-di-GMP can be bound by some diguanylate cyclase enzymes to allosterically repress its own synthesis (Chan *et al.*, 2004; Wassmann *et al.*, 2007;). The other known protein targets are *G. xylinus* cellulose synthase (Ross *et al.*, 1985, 1987) and other PilZ domain proteins (Ryjenkov *et al.*, 2006), and *P. aeruginosa* PelD protein (Lee *et al.*, 2007). It was found in a recent study that cyclic-di-GMP, in many bacterial species, is sensed by a riboswitch class in mRNA that controls the expression of genes involved in numerous fundamental cellular processes (Sudarsan *et al.*, 2008). Although the control mechanisms by cyclic-di-GMP for the development of *S. aureus* and *P. aeruginosa* biofilms are not well understood, and previous studies suggested that cyclic-di-GMP bound to its target proteins for the regulation (Ross *et al.*, 1985; Ryjenkov *et al.*, 2006; Lee *et al.*, 2007;), it is interesting to examine whether riboswitches are involved in the cyclic-di-GMP signaling pathways for biofilm formation. Because the analogous cyclic nucleotides measured in this study expressed similar suppression activities on the biofilm formation and it seems that the differences were not qualitative but quantitative, we speculate that these compounds can function like cyclic-di-GMP.

In a previous study, Karaolis *et al.* (2005) demonstrated that a high concentration of cyclic-di-GMP added extracellularly suppressed the biofilm formation of *S. aureus*. We found in the present study that low concentrations of cyclic-di-GMP enhanced the *S. aureus* biofilm formation and high concentrations of extracellular cyclic-di-GMP suppressed the formation of gram-negative *P. aeruginosa* biofilm as well as *S. aureus* biofilm. Because cyclic-di-GMP is an intracellular second messenger to regulate wide-ranging physiological changes, it is rational to speculate that extracellular cyclic-di-GMP penetrates through bacterial cells and expresses its biological activities in the cells, although evidences showing that cyclic-di-GMP penetrates across the membrane have not yet been reported. If it is true, its analogous compounds also likely penetrated through both gram-positive and gram-negative bacterial cells in a similar way, and suppressed bacterial biofilm formation. However, it is also possible that the regulation by extracellular cyclic-di-GMP and its analogs is mediated by a cell surface receptor.

Control of the bacterial biofilm formation *in vivo* and *in vitro* with synthetic compounds may be indispensable for prevention of hospital-associated infections, because a large number of such infections are associated with bacterial biofilms formed on the surface of artificial devices that are introduced into patient bodies. The present study demonstrated that the activities of cyclic-di-GMP and cyclic-GpGps were higher than those of cyclic-GpAp and cyclic-GpIp, suggesting a possibility for the development of effective agents that interfere with the formation of bacterial biofilms.

## Statement

Re-use of this article is permitted in accordance with the Terms and Conditions set out at http://www3.interscience.wiley.com/authorresources/onlineopen.html
